# Non-Metricity in Information Geometry

**DOI:** 10.3390/e28040447

**Published:** 2026-04-14

**Authors:** Tatsuaki Wada, Antonio Maria Scarfone

**Affiliations:** 1 Region of Electrical and Electronic Systems Engineering, Ibaraki University, 4-12-1 Nakanarusawa-cho, Hitachi-shi 316-8511, Japan; 2 Istituto dei Sistemi Complessi, Consiglio Nazionale delle Ricerche (ISC-CNR), c/o Politecnico di Torino, Corso Duca degli Abruzzi, 24, 10129 Torino, Italy; antonio.scarfone@polito.it; 3 Istituto Nazionale di Fisica Nucleare (INFN), Sezione di Torino, Via Pietro Giuria, 1, 10125 Torino, Italy

**Keywords:** α connection, gradient flow, Weyl geometry, non-metricity, information geometry

## Abstract

The importance of non-metricity in information geometry is described through studies on gradient flow from the perspective of Weyl geometry. The Amari–Centsov tensor $C_{kij}$ is the non-metricity tensor with respect to the α connection $\nabla^{\left(\alpha \right)}$ and Fisher metric $g_{ij}$. We derive the explicit expression of the α connection based on the non-metricity $\nabla_{k}^{\left(\alpha \right)}g_{ij}=\alpha C_{kij}$. The scalar field obtained from a potential function in information geometry plays a key role and characterizes Weyl’s gauge field, Weyl’s non-metricity, and the rate of the potential function during the associated gradient flow.

## 1. Introduction

Information geometry (IG) [1,2,3] provides a powerful, interdisciplinary framework that bridges information theory, statistics, and physics by applying differential geometry to the space of probability distributions. The theoretical information structure in IG is called α geometry. Let us begin with a brief explanation of α geometry. IG has been developed by mainly considering a parametrized statistical model

(1) $$\begin{array}{c} p_{\theta }\left(x\right)=\exp\left[\theta^{i}F_{i}\left(x\right)-\Psi \left(\theta \right)\right], \end{array}$$

which is called an exponential probability distribution function (pdf). Here the θ potential Ψ(θ) is determined from the normalization of $p_{\theta }\left(x\right)$ as $\Psi \left(\theta \right)=\ln\left[\int dx\exp(\theta^{i}F_{i}\left(x\right))\right]$, i.e., the cumulant generating function. The expectation coordinates $\left\{\eta_{i}\right\}$ are obtained from Ψ(θ) as

(2) $$\begin{array}{c} \eta_{i}=\frac{\partial \Psi (\theta )}{\partial \theta^{i}}, \end{array}$$

which are the dual functions of the natural coordinates $\left\{\theta^{i}\right\}$. The Legendre dual potential function $\Psi^{⋆}\left(\eta \right):=\theta^{i}\;\eta_{i}-\Psi \left(\theta \right)$ is the (negative sign of) entropic function

(3) $$\begin{array}{c} \Psi^{⋆}\left(\eta \right)=E_{p_{\theta }}\left[\ln p_{\theta }\left(x\right)\right], \end{array}$$

and the natural coordinates satisfy $\theta^{i}=\partial \Psi^{⋆}\left(\eta \right)/\partial \eta_{i}$.

By assuming Ψ(θ) or $\Psi^{⋆}\left(\eta \right)$ is convex, the positive definite matrices $g_{ij}\left(\theta \right)$ and $g^{ij}\left(\eta \right)$ are obtained from the Hessian matrices as

(4) $$\begin{array}{c} g_{ij}\left(\theta \right)=\frac{\partial^{2}\Psi \left(\theta \right)}{\partial \theta^{i}\partial \theta^{j}},\;g^{ij}\left(\eta \right)=\frac{\partial^{2}\Psi^{⋆}\left(\eta \right)}{\partial \eta_{i}\partial \eta_{j}}, \end{array}$$

respectively. These matrices satisfy the relation $g^{ij}\left(\eta \right)\;g_{jk}\left(\theta \right)=\delta_{k}^{i}$, where $\delta_{k}^{i}$ denotes Kronecker’s delta.

As one-parameter (α) extensions of the Levi–Civita connection $\nabla^{\left(0\right)}$, the α connection $\nabla^{\left(\alpha \right)}$, and its dual $\nabla^{⋆(\alpha )}$ are introduced [1,2,3] and defined by their coefficients

(5) $$\begin{array}{c} {\Gamma^{\left(\alpha \right)}}_{ijk}\left(\theta \right):=\frac{\left(1-\alpha \right)}{2}C_{ijk}\left(\theta \right),\;\Gamma^{⋆(\alpha )\;ijk}\left(\eta \right):=\frac{\left(1+\alpha \right)}{2}C^{⋆ijk}\left(\eta \right), \end{array}$$

respectively. Here $C_{ijk}\left(\theta \right)$ and its dual $C^{⋆ijk}\left(\eta \right)$ are called Amari–Centsov tensors defined by

(6) $$\begin{array}{c} C_{ijk}\left(\theta \right):=\frac{\partial^{3}\Psi \left(\theta \right)}{\partial \theta^{i}\partial \theta^{j}\partial \theta^{k}},\;C^{⋆ijk}\left(\eta \right):=\frac{\partial^{3}\Psi^{⋆}\left(\eta \right)}{\partial \eta_{i}\partial \eta_{j}\partial \eta_{k}}, \end{array}$$

respectively. It is known that the Fisher metric $g_{ij}\left(\theta \right)$ and Amari–Centsov tensor $C_{ijk}\left(\theta \right)$ are characterized by Centsov’s theorem [4]. A coordinate system in which all connection coefficients become zero is called affine coordinate system. It is well-known that α=±1 plays a central role [1,2], in which the connection coefficients ${\Gamma^{\left(1\right)}}_{ijk}\left(\theta \right)$ or $\Gamma^{⋆(-1)\;ijk}\left(\eta \right)$ vanish as shown from the factor (1−α) or (1+α) in (5). Hence the θ coordinates (η-coordinates) are affine coordinates for the connection $\nabla^{\left(1\right)}$ ($\nabla^{⋆(-1)}$). This feature is known as the dually flat structure in IG.

Incidentally, for a given metric $g_{jk}$ and an independent connection ∇, the non-metricity tensor

(7) $$\begin{array}{c} Q_{ijk}:=\nabla_{i}\;g_{jk}, \end{array}$$

characterizes the deviation from the metricity condition $\nabla_{i}\;g_{jk}=0$. Unlike in the case of metricity, raising or lowering the indices of a vector $v^{i}$ (or tensor) under the covariant derivative ∇ is not straightforward. Since

(8) $$\begin{array}{c} \nabla_{k}v_{i}=\nabla_{k}\left(g_{ij}v^{j}\right)=\left(\nabla_{k}g_{ij}\right)v^{j}+g_{ij}\nabla_{k}v^{j}=Q_{kij}v^{j}+g_{ij}\nabla_{k}v^{j}, \end{array}$$

it follows that

(9) $$\begin{array}{c} g_{ij}\nabla_{k}v^{j}=\nabla_{k}v_{i}-Q_{kij}v^{j}. \end{array}$$

In other words, a connection ∇ and a metric *g* are non-commutatative. For a given velocity $u^{i}$, the acceleration $a^{i}$ is defined by

(10) $$\begin{array}{c} a^{i}:=u^{k}\nabla_{k}u^{i}, \end{array}$$

and the *anomalous acceleration* $\tilde{a_{i}}$ is defined by

(11) $$\begin{array}{cc} {\tilde{a}}_{i} & :=u^{k}\nabla_{k}u_{i}=u^{k}\nabla_{k}\left(g_{ij}u^{j}\right)=g_{ij}u^{k}\nabla_{k}u^{j}+u^{k}u^{j}\nabla_{k}g_{ij}=g_{ij}a^{j}+u^{k}u^{j}Q_{kij}. \end{array}$$

Hence lowering the index of the acceleration $a_{i}:=g_{ij}a^{i}$ and the anomalous acceleration ${\tilde{a}}_{i}$ are different when the non-metricity tensor $Q_{kij}$ are non-zero.

In IG, because both curvature and torsion are zero in dually flat spaces, non-metricity must play a role. However, as far as we know, the technical word “non-metricity” is almost never used in the literature on IG, except by Hirano and Nagahama [5]. They pointed out in their Section 2.3 [5] that the non-metricity tensor associated with the α connection $\nabla^{\left(\alpha \right)}$ is proportional to the Amari–Centsov tensor $C_{ijk}\left(\theta \right)$, i.e.,

(12) $$\begin{array}{c} Q_{ijk}^{\left(\alpha \right)}\left(\theta \right):=\nabla_{i}^{\left(\alpha \right)}\;g_{jk}\left(\theta \right)=\alpha \;C_{ijk}\left(\theta \right). \end{array}$$

Here, the definition (7) of a non-metricity tensor is different (opposite in sign) from the definition in Ref. [5]. It is well-known in the field of IG [2,3] that the Amari–Centsov tensor $C_{ijk}\left(\theta \right)$ is important for defining the α connection $\nabla^{\left(\alpha \right)}$ with its coefficients

(13) $$\begin{array}{c} {\overset{\left(\alpha \right)}{\Gamma^{i}}}_{jk}\left(\theta \right):={\overset{\mathrm{LC}}{\Gamma^{i}}}_{jk}\left(\theta \right)-\frac{\alpha }{2}\;g^{i\ell }\left(\theta \right)C_{\ell jk}\left(\theta \right), \end{array}$$

where ${\overset{\mathrm{LC}}{\Gamma^{i}}}_{jk}\left(\theta \right)$ denotes the coefficients of the Levi–Civita connection $\overset{\mathrm{LC}}{\nabla }$ with respect to (w.r.t.) the Fisher metric $g_{ij}\left(\theta \right)$. However, in the conventional explanations in IG, the α connection $\nabla^{\left(\alpha \right)}$ is introduced by definition (13). We shall explore the importance of the non-metricity in IG in Section 4.

Non-metricity is also a key ingredient in Weyl geometry [6,7,8], which is a generalization of Riemannian geometry. In Weyl geometry, a parallel transported vector changes its length, which is characterized by *Weyl’s non-metricity*

(14) $$\begin{array}{c} Q_{ijk}^{w}\left(\theta \right):={\overset{W}{\nabla }}_{i}\;g_{jk}\left(\theta \right)=\omega_{i}\left(\theta \right)\;g_{jk}\left(\theta \right),\;{\overset{W}{\nabla }}_{i}\;g^{jk}\left(\theta \right)=-\omega_{i}\left(\theta \right)\;g^{jk}\left(\theta \right), \end{array}$$

where $\omega_{i}\left(\theta \right)$ is called a Weyl gauge field, and $\overset{W}{\nabla }$ is Weyl’s connection. The coefficients ${\overset{W}{\Gamma^{i}}}_{jk}\left(\theta \right)$ of Weyl’s connection $\overset{W}{\nabla }$ are obtained [7,8] from Weyl’s non-metricity (14) as follows:

(15) $$\begin{array}{c} {\overset{W}{\Gamma^{i}}}_{jk}\left(\theta \right)={\overset{\mathrm{LC}}{\Gamma^{i}}}_{jk}\left(\theta \right)-\frac{1}{2}\left\{\delta_{j}^{i}\;\omega_{k}\left(\theta \right)+\delta_{k}^{i}\;\omega_{j}\left(\theta \right)\;-g^{i\ell }\left(\theta \right)\;\omega_{\ell }\left(\theta \right)\;g_{jk}\left(\theta \right)\right\}. \end{array}$$

It is worth noting that the non-metricity tensor $Q_{ijk}^{\left(\alpha \right)}\left(\theta \right)$ in (12) and $Q_{ijk}^{w}\left(\theta \right)$ in (14) are different. The former is a tensor w.r.t. the α connection $\nabla^{\left(\alpha \right)}$ and proportional to $C_{ijk}\left(\theta \right)$. In contrast, the latter is a tensor w.r.t. the Weyl connection $\overset{W}{\nabla }$ and proportional to $g_{jk}\left(\theta \right)$.

Together with our collaborators, gradient flow [9,10,11,12] in IG had been studied from different perspectives and is related to some different fields of physics: anisotropic Huygens’ equations in geometric optics [13]; analytical mechanics and black hole thermodynamics [14]. Furthermore Ref. [15] studied gradient flow w.r.t. the divergence functions from the motion of a null (light-like) particle in curved spacetime. It was the first study [16] of gradient flow from the perspective of Weyl geometry. Ref. [17] studied gradient flow in IG in spacetime and showed Weyl’s gauge symmetry. Research focusing on the mathematical and scientific structures common to different fields lies at the heart of SUURI engineering. Prof. Amari is widely recognized as the founder of SUURI engineering, and IG is a field of SUURI engineering. Many papers and books concerning the foundation of IG have been published, but it is impossible to list them all, as they are too many. Caticha [18] developed IG as a foundation for physics, particularly in the fields of statistical inference and thermodynamics. We would also like to develop a physical foundation of IG.

In this paper, we show the importance of non-metricity in IG through some studies on gradient flow from the perspective of Weyl geometry and α geometry. The next section gives a quick review of gradient flow equations in IG. In Section 3, we first briefly review Weyl geometry, Weyl’s symmetry, and Weyl proper time, all of which are characterized in terms of a Weyl gauge field $\omega_{i}$. Then in Section 3.1, we show that gradient flow equations are obtained from the variational principle of the Weyl symmetric action $S_{a}$ in (30). The importance of the scalar field $\eta^{2}\left(\theta \right)$ is emphasized from some different points of view. In Section 4, we show the importance of non-metricity in IG. After confirming the non-metricity relation of (12), we show that the second term in (13) represents a disformation tensor. In Section 4.1, we derive the coefficients ${\overset{\left(\alpha \right)}{\Gamma^{i}}}_{jk}\left(\theta \right)$ of the α connection $\nabla^{\left(\alpha \right)}$ based on the non-metricity tensor (12) characterized by the Amari–Centsov tensor. Section 4.2 shows that gradient flow equations are characterized by autoparallel equations in IG. The final Section 5 is devoted to our conclusion and perspective. Appendix A shows the derivation of the autoparallel equations in α geometry.

Throughout this paper, we use the index notations commonly used in general relativity. All indices run from 1 to *N*, which represent the dimensions of an appropriate space.Unless otherwise stated, the Einstein summation convention is used.

## 2. Gradient-Flow Equations

We briefly explain gradient flow equations [9,10,11,12] in IG. For the sake of simplicity, we consider gradient flow equations w.r.t. the θ potential Ψ(θ), i.e.,

(16) $$\begin{array}{c} \frac{d\theta^{i}}{dt}=g^{ij}\left(\theta \right)\;\frac{\partial \Psi (\theta )}{\partial \theta^{j}}, \end{array}$$

in the θ-coordinate system. The right-hand side expresses the natural gradient [19] w.r.t. the Fisher metric. By using (4), the left-hand side of (16) is rewritten as

(17) $$\begin{array}{cc} \frac{d\theta^{i}}{dt} & =\underset{g^{ij}\left(\theta \right)}{\underset{︸}{\frac{\partial \theta^{i}}{\partial \eta_{j}}}}\frac{d\eta_{j}}{dt}=g^{ij}\left(\theta \right)\frac{d\eta_{j}}{dt}, \end{array}$$

and using (2) the right-hand side of (16) leads to $g^{ij}\left(\theta \right)\;\eta_{j}$. Consequently, the gradient flow Equation (16) in the θ-coordinate system are equivalent to linear differential equations

(18) $$\begin{array}{c} \frac{d\eta_{i}\left(t\right)}{dt}=\eta_{i}\left(t\right), \end{array}$$

in the η-coordinate system. This linearization is a merit due to the dually flat structure [1,2,3] in IG.

The other set (or the dual) of gradient flow equations are given by

(19) $$\begin{array}{c} \frac{d\eta_{i}}{dt}=-g_{ij}\left(\eta \right)\;\frac{\partial \Psi^{⋆}\left(\eta \right)}{\partial \eta_{j}}, \end{array}$$

in the η-coordinate system. Similarly, they are equivalent to the linear differential equations

(20) $$\begin{array}{c} \frac{d\theta^{i}\left(t\right)}{dt}=-\theta^{i}\left(t\right), \end{array}$$

in the θ-coordinate system In previous works [13,14,16], gradient-flow equations w.r.t. the θ- or η-potential functions were considered. It is worth emphasizing that the two sets of differential Equations (16) and (19) describe different processes in general [13,14]. In addition, the evolutional parameter *t* in the gradient flow Equations (16) and (19) is a non-affine parameter.

Gradient flow (16) and (19) can be related to Hamilton flow characterized by the following Hamiltonians

(21) $$\begin{array}{c} H\left(\theta ,\eta \right)=\frac{1}{2}g^{ij}\left(\theta \right)\eta_{i}\eta_{j}-\frac{1}{2}\eta^{2}\left(\theta \right),\;H\left(\eta ,-\theta \right)=\frac{1}{2}g_{ij}\left(\eta \right)\theta^{i}\theta^{j}-\frac{1}{2}\theta^{2}\left(\eta \right), \end{array}$$

respectively. Here $\eta^{2}\left(\theta \right)$ and $\theta^{2}\left(\eta \right)$ are given by

(22) $$\begin{array}{c} \eta^{2}\left(\theta \right):=g^{ij}\left(\theta \right)\frac{\partial \Psi (\theta )}{\partial \theta^{i}}\frac{\partial \Psi (\theta )}{\partial \theta^{j}},\;\theta^{2}\left(\eta \right):=g_{ij}\left(\eta \right)\frac{\partial \Psi^{⋆}\left(\eta \right)}{\partial \eta_{i}}\frac{\partial \Psi^{⋆}\left(\eta \right)}{\partial \eta_{j}}, \end{array}$$

respectively. Note also that H(θ,η) and H(η,−θ) are related through the canonical transformation $\left(\theta^{i},\eta_{i}\right)$ to $\left(\eta_{i},-\theta^{i}\right)$ [14].

It is worth noting that the scalar field $\eta^{2}\left(\theta \right)$ characterizes the rate of the θ potential, since it is related to the θ-potential function Ψ(θ) as follows:

(23) $$\begin{array}{cc} \frac{d\Psi (\theta )}{dt} & =\frac{\partial \Psi (\theta )}{\partial \theta^{i}}\underset{g^{ij}\left(\theta \right)\partial \Psi \left(\theta \right)/\partial \theta^{j}}{\underset{︸}{\frac{d\theta^{i}}{dt}}}=g^{ij}\left(\theta \right)\underset{\eta_{i}}{\underset{︸}{\frac{\partial \Psi (\theta )}{\partial \theta^{i}}}}\underset{\eta_{j}}{\underset{︸}{\frac{\partial \Psi (\theta )}{\partial \theta^{j}}}}=\eta^{2}\left(\theta \right). \end{array}$$

where the relations (2) and (16) are used. Similarly, the scalar field $\theta^{2}\left(\eta \right)$ characterizes the rate of the η potential as

(24) $$\begin{array}{c} \frac{d\Psi^{⋆}\left(\eta \right)}{dt}=-\theta^{2}\left(\eta \right). \end{array}$$

Since $-\Psi^{⋆}\left(\eta \right)$ is the entropy S(η) w.r.t. the pdf $p_{\theta }\left(x\right)$, the scalar field $\theta^{2}\left(\eta \right)$ characterizes the rate of the entropy dS(η)/dt in the gradient flow (19).

## 3. Weyl Geometry

As briefly mentioned in the Introduction, Weyl geometry is a generalization of Riemannian geometry based on the gauge field $\omega_{i}\left(x\right)$, which specifies the non-metricity (14). The Weyl connection (15) is an extension of the Levi–Civita connection with a given gauge field $\omega_{i}\left(x\right)$. A parallel transported vector changes in length according to the non-metricity (14). When $\omega_{i}\left(x\right)=0$, Weyl geometry reduces to Riemannian geometry.

Weyl [6,20] introduced the concept of *Weyl’s symmetry* and the equivalent classes of $\left(g_{jk},\omega_{i}\right)$. An action or equation, which consists of a metric $g_{jk}\left(x\right)$ and a scalar field φ(x), has Weyl’ symmetry, if it is invariant under the Weyl transformation

(25) $$\begin{array}{c} g_{jk}\left(x\right)\to \Omega^{2}\left(x\right)\;g_{jk}\left(x\right),\;\varphi \left(x\right)\to \Omega^{-1}\left(x\right)\;\varphi \left(x\right),\;\omega_{i}\left(x\right)\to \omega_{i}\left(x\right)+\partial_{i}\ln\Omega^{2}\left(x\right), \end{array}$$

which makes a local rescaling of $g_{jk}\left(x\right)$, φ(x) and the Weyl gauge field $\omega_{i}\left(x\right)$ with a positive scalar function Ω(x). It is worth noting that the Weyl connection (15) is invariant under the Weyl transformation (25).

Each equivalent class $\left(g_{jk}\left(x\right),\omega_{i}\left(x\right)\right)$ is related to another class by the Weyl gauge transformation (25). Fixing the gauge incolves choosing a certain equivalent class of $\left(g_{jk}\left(x\right),\omega_{i}\left(x\right)\right)$. A special class of $\left(g_{jk}\left(x\right),\omega_{i}\left(x\right)=0\right)$ is called the Einstein gauge, in which non-metricity (14) reduces to metricity, and consequently Weyl geometry reduces to Riemannian geometry.

Next, for an arbitrary time parameter λ, the most general form [7,17] of the autoparallel equations in Weyl geometry is given by

(26) $$\begin{array}{c} u^{k}\left(\lambda \right){\overset{W}{\nabla }}_{k}u^{i}\left(\lambda \right)=\frac{1}{2u^{2}\left(\lambda \right)}\left(\frac{du^{2}\left(\lambda \right)}{d\lambda }-\omega_{k}\left(\theta \right)u^{k}\left(\lambda \right)u^{2}\left(\lambda \right)\right)u^{i}\left(\lambda \right), \end{array}$$

where $u^{i}\left(\lambda \right):=dx^{i}/d\lambda$. For a given Weyl gauge field $\omega_{i}\left(x\right)$ and tangent vector $u^{j}\left(\tau \right):=dx^{j}\left(\tau \right)/d\tau$, parameter τ is called *Weyl proper time* [21] if it satisfies

(27) $$\begin{array}{c} \frac{du^{2}\left(\tau \right)}{d\tau }=u^{2}\left(x\right)\;\omega_{i}\left(x\right)u^{i}\left(\tau \right), \end{array}$$

where $u^{2}\left(\tau \right):=g_{jk}\left(x\right)u^{j}\left(\tau \right)u^{k}\left(\tau \right)$. Note that the tangent vector $u^{j}\left(x\right)$ depends on parameter τ, and the right-hand side of (26) becomes zero for Weyl proper time τ.

Having briefly explained Weyl geometry, the next subsection reconsiders gradient flow in IG from the perspective of Weyl geometry.

### 3.1. The Gradient-Flow in Weyl Geometry

We introduce the infinitesimal line element ds of the arc-length parameter *s* in θ space as

(28) $$\begin{array}{c} ds^{2}:=g_{ij}\left(\theta \right)d\theta^{i}d\theta^{j}. \end{array}$$

Then infinitesimal line element dτ of Weyl proper time τ is related [16,17] as

(29) $$\begin{array}{c} d\tau =\sqrt{\eta^{2}\left(\theta \right)}\;ds, \end{array}$$

where the positive scalar function $\eta^{2}\left(\theta \right)$ is defined in (22). Then the action integral is given [17] by

(30) $$\begin{array}{c} S_{a}=\int d\tau =\int \sqrt{\eta^{2}\left(\theta \right)}\;ds=\int \sqrt{\eta^{2}\left(\theta \right)g_{ij}\left(\theta \right)d\theta^{i}d\theta^{j}}=:\int L\left(\theta ,\frac{d\theta }{d\lambda }\right)d\lambda , \end{array}$$

where L(θ,dθ/dλ) denotes the Lagrangian for arbitrary time parameter λ. It is worth noting that action $S_{a}$ is invariant to the reparametrization of λ. Therefore it is crucial to specify $S_{a}$. Later we specify the time parameter *t* in order to make the Lagrangian L(θ,dθ/dt) describe gradient flow Equation (16). Note that $\eta^{2}\left(\theta \right)$ should not be confused with $\eta^{2}:=g^{ij}\left(\theta \right)\eta_{i}\eta_{j}$. They have different functional dependences but the same value after taking the variation of $S_{a}$.

In addition, action $S_{a}$ is invariant to the local rescaling of

(31) $$\begin{array}{c} ds\to \Omega \left(\theta \right)ds,\;\mathrm{and}\;\eta^{2}\left(\theta \right)\to \frac{\eta^{2}\left(\theta \right)}{\Omega^{2}\left(\theta \right)}, \end{array}$$

with a positive scalar function Ω(θ). The square root form of the Lagrangian in (30) involves a technical difficulty, especially for a massless particle, which corresponds to the case $\eta^{2}\left(\theta \right)=0$. It is convenient to introduce an einbein field e(λ) [22] as then the Lagrangian becomes

(32) $$\begin{array}{c} L\left(\theta ,\frac{d\theta }{d\lambda }\right)=\frac{1}{2e(\lambda )}g_{ij}\left(\theta \right)\frac{d\theta^{i}}{d\lambda }\frac{d\theta^{j}}{d\lambda }+\frac{e(\lambda )}{2}\eta^{2}\left(\theta \right). \end{array}$$

Here the expression of einbein e(λ) is obtained from the Euler–Lagrange (EL) equation ∂L/∂e(λ)=0 w.r.t. einbein e(λ) as follows:

(33) $$\begin{array}{c} e\left(\lambda \right)=\frac{1}{\sqrt{\eta^{2}\left(\theta \right)}}\sqrt{g_{ij}\left(\theta \right)\frac{d\theta^{i}}{d\lambda }\frac{d\theta^{j}}{d\lambda }}=\frac{1}{\sqrt{\eta^{2}\left(\theta \right)}}\frac{ds}{d\lambda }. \end{array}$$

Note that if we substitute this expression of e(λ) into (32), the square root form in (30) is recovered. One readily finds from (33) that any set of parameters λ and $\lambda^{\prime }$ is related as

(34) $$\begin{array}{c} e\left(\lambda \right)d\lambda =e\left(\lambda^{\prime }\right)d\lambda^{\prime }. \end{array}$$

Now let us choose parameter *t* such that the einbein e(t) in (33) is unity, i.e.,

(35) $$\begin{array}{c} e\left(t\right)=\frac{1}{\sqrt{\eta^{2}\left(\theta \right)}}\frac{ds}{dt}=1, \end{array}$$

which means that $ds=\sqrt{\eta^{2}\left(\theta \right)}\;dt$. Combining this relation with (29), we have $d\tau =\eta^{2}\left(\theta \right)\;dt$. Then the canonical momenta $\eta_{i}:=\partial L/\partial \left(d\theta^{i}/dt\right)$ and EL equations of Lagrangian (32) w.r.t. $\theta^{i}\left(t\right)$ become

(36) ηi=gij(θ)dθjdt,(37)dηidt=12∂gjk(θ)∂θidθjdtdθkdt+12∂η2(θ)∂θi.

The relations (36) are equivalent to the gradient flow equations (16). By differentiating both sides of $\eta^{2}\left(\theta \right)$ in (22), we have

$$\begin{array}{cc} \frac{1}{2}\frac{\partial \eta^{2}\left(\theta \right)}{\partial \theta^{i}}\; & =\frac{1}{2}\frac{\partial g^{jk}\left(\theta \right)}{\partial \theta^{i}}\underset{\eta_{j}}{\underset{︸}{\frac{\partial \Psi (\theta )}{\partial \theta^{j}}}}\underset{\eta_{k}}{\underset{︸}{\frac{\partial \Psi (\theta )}{\partial \theta^{k}}}}+g^{jk}\left(\theta \right)\underset{g_{ij}\left(\theta \right)}{\underset{︸}{\frac{\partial^{2}\Psi \left(\theta \right)}{\partial \theta^{i}\partial \theta^{j}}}}\underset{\eta_{k}}{\underset{︸}{\frac{\partial \Psi (\theta )}{\partial \theta^{k}}}}, \end{array}$$

which are rewritten by using (36) as follows:

(38) $$\begin{array}{cc} \frac{1}{2}\frac{\partial \eta^{2}\left(\theta \right)}{\partial \theta^{i}}\; & =\frac{1}{2}\underset{-\partial_{i}g_{km}\left(\theta \right)g^{jk}\left(\theta \right)}{\underset{︸}{\frac{\partial g^{jk}\left(\theta \right)}{\partial \theta^{i}}g_{km}\left(\theta \right)}}\frac{d\theta^{m}}{dt}g_{j\ell }\left(\theta \right)\frac{d\theta^{\ell }}{dt}+\underset{\delta_{i}^{k}}{\underset{︸}{g^{jk}\left(\theta \right)g_{ij}\left(\theta \right)}}\eta_{k}\\ \; & =-\frac{1}{2}\frac{\partial g_{km}\left(\theta \right)}{\partial \theta^{i}}\underset{{\delta^{k}}_{\ell }}{\underset{︸}{g^{jk}\left(\theta \right)g_{j\ell }\left(\theta \right)}}\frac{d\theta^{m}}{dt}\frac{d\theta^{\ell }}{dt}+\eta_{i}=-\frac{1}{2}\frac{\partial g_{jk}\left(\theta \right)}{\partial \theta^{i}}\frac{d\theta^{j}}{dt}\frac{d\theta^{k}}{dt}+\eta_{i}. \end{array}$$

Substituting this into (37) leads to linearized Equation (18). In this way, we can derive both gradient flow Equations (16) and (18) from the Lagrangian (32). We hence show that the variational principle holds for gradient flow in IG.

At this point, some comments are required. Firstly, the scalar function $\eta^{2}\left(\theta \right)$ is key in action $S_{a}$ in (30), which is invariant to local rescaling (31).

Secondly, in previous work [16], we showed that, for gradient flow Equation (16) in θ space, the Weyl gauge field is

(39) $$\begin{array}{c} \omega_{i}\left(\theta \right)=-\partial_{i}\ln\eta^{2}\left(\theta \right), \end{array}$$

in which $\omega_{i}\left(\theta \right)$ is the *i*-th component of the gradient of scalar function $-\ln\eta^{2}\left(\theta \right)$, i.e., this is Weyl integrable geometry. Again, quantity $\eta^{2}\left(\theta \right)$ is key in Weyl gauge $\omega_{i}\left(\theta \right)$, which characterizes Weyl’s non-metricity (14).

Thirdly, as mentioned in the Introduction, quantity $\eta^{2}\left(\theta \right)$ characterizes rate (23) of θ-potential function Ψ(θ) during gradient flow (16). Rearranging the well-known relation $d\Psi \left(\theta \right)=\left(\partial \Psi \left(\theta \right)/\partial \theta^{i}\right)d\theta^{i}=\eta_{i}d\theta^{i}$ and using (23), it follows that

(40) $$\begin{array}{c} 0=\eta_{i}d\theta^{i}-\eta^{2}\left(\theta \right)dt=:\omega_{\mathrm{IG}}, \end{array}$$

which has the same form as the Poincaré–Cartan form $\omega_{\mathrm{PC}}:=p_{i}dq^{i}-E\left(t\right)dt$ in analytical mechanics. Ref. [15] discussed complete integrability on the Pfaffian Equation (40) and obtained the null Hamiltonian describing gradient flow (16) in the following form: $H\left(\theta ,\eta \right)=\sqrt{g^{ij}\left(\theta \right)\eta_{i}\eta_{j}}-\sqrt{\eta^{2}\left(\theta \right)}$, which is the square root version of H(θ,η) in (21). For details, please refer to Section 3 in [15]. As Cartan [23] emphasized the importance of $\omega_{\mathrm{PC}}$, in our case, $\omega_{\mathrm{IG}}$ is important for describing gradient flow. By taking the exterior derivative of $-\omega_{\mathrm{IG}}$, we have

(41) $$\begin{array}{c} -d\omega_{\mathrm{IG}}=d\theta^{i}\wedge d\eta_{i}+d\eta^{2}\left(\theta \right)\wedge dt, \end{array}$$

which is an extension of the standard symplectic form $-d\left(\eta_{i}d\theta^{i}\right)=d\theta^{i}\wedge d\eta_{i}$. Here again, quantity $\eta^{2}\left(\theta \right)$ plays an important role. In standard symplectic geometry, the value of any Hamilton function $H^{\mathrm{sym}}=H^{\mathrm{sym}}\left(\theta ,\eta \right)$ is a constant of motion (the total energy of the system). Hence any $H^{\mathrm{sym}}\left(\theta ,\eta \right)$ cannot describe a dissipative system.

Finally, let us focus on the Lagrangian (32) for choosing the time parameter *t*. Since the canonical momenta is $\eta_{i}=g_{ij}\left(\theta \right)\left(d\theta^{j}/dt\right)$, the associated Hamiltonian is

(42) $$\begin{array}{c} H\left(\theta ,\eta \right):=\eta_{i}\frac{d\theta^{i}}{dt}-L\left(\theta ,\frac{d\theta }{dt}\right)=\frac{1}{2}g^{ij}\left(\theta \right)\eta_{i}\eta_{j}-\frac{1}{2}\eta^{2}\left(\theta \right), \end{array}$$

which is the Hamiltonian H(θ,η) in (21).

### 3.2. Relation to the α-Connection

Here we discuss the relation [16] between the Weyl connection and the α connection when they describe the same gradient flow Equation (16).

Gradient flow Equation (16) are equivalently expressed [16] in terms of $u^{i}\left(t\right)=d\theta^{i}/dt$ as autoparallel equations

(43) $$\begin{array}{c} u^{k}\left(t\right)\nabla_{k}^{\left(-1\right)}u^{i}\left(t\right)=u^{i}\left(t\right), \end{array}$$

in α geometry with α=−1 connection $\nabla^{\left(-1\right)}$.

Next, let us choose the Weyl gauge field $\omega^{i}\left(\theta \right)=-\partial \ln\eta^{2}\left(\theta \right)/\partial \theta^{i}$ as (37) and the time parameter *t*, which satisfies (35). Then it follows that $u^{2}\left(t\right):=u_{i}\left(t\right)u^{i}\left(t\right)=\eta^{2}\left(\theta \right)$, where $u_{i}\left(t\right):=g_{ij}\left(\theta \right)u^{j}\left(t\right)=\eta_{i}$. The corresponding Weyl autoparallel Equation (26) are

(44) $$\begin{array}{c} u^{k}\left(t\right){\overset{W}{\nabla }}_{k}u^{i}\left(t\right)=-\omega_{k}u^{k}\left(t\right)u^{i}\left(t\right), \end{array}$$

which have a clear physical interpretation. The left-hand side represents acceleration w.r.t. velocity $u^{i}\left(t\right)=d\theta^{i}/dt$. The right-hand side represents force proportional to velocity $u^{i}\left(t\right)$, whose proportional factor is a function of velocity $u^{k}\left(t\right)$ and Weyl gauge field $\omega_{k}\left(\theta \right)$.

In addition, by applying the Weyl connection to both sides of (36) and contracting with $u^{k}\left(t\right)$, we have

(45) $$\begin{array}{c} u^{k}\left(t\right){\overset{W}{\nabla }}_{k}\eta_{i}=u^{k}\left(t\right)u^{j}\left(t\right)\underset{\omega_{k}\left(\theta \right)g_{ij}\left(\theta \right)}{\underset{︸}{{\overset{W}{\nabla }}_{k}g_{ij}\left(\theta \right)}}+g_{ij}\left(\theta \right)\underset{-\omega_{k}\left(\theta \right)u^{k}\left(t\right)u^{i}\left(t\right)}{\underset{︸}{u^{k}\left(t\right){\overset{W}{\nabla }}_{k}u^{j}\left(t\right)}}=0, \end{array}$$

which means that $\eta_{i}$ is parallel-transported along a trajectory $\theta^{i}=\theta^{i}\left(t\right)$ of Equation (44). In other words, recalling that the right-hand side of (45) expresses anomalous acceleration (11), we see that Equation (45) states that the anomalous acceleration is zero. In the Weyl gauge $\left(g_{jk}\left(\theta \right),\omega_{i}=-\partial_{i}\ln\eta^{2}\left(\theta \right)\right)$, Equations (44) and (45) characterize gradient flow (16).

As shown in [16,17], gradient flow (16) is equivalently described bynboth the autoparalell Equation (44) in Weyl geometry and by the autoparallel Equation (43). Consequently, Weyl connection $\overset{W}{\nabla }$ is related to the α connection of α=−1 as

(46) $$\begin{array}{c} {\overset{W}{\Gamma^{i}}}_{jk}\left(\theta \right)\;u^{j}\left(t\right)u^{k}\left(t\right)={{\Gamma^{\left(-1\right)}}^{i}}_{jk}\left(\theta \right)\;u^{j}\left(t\right)u^{k}\left(t\right)+\frac{\partial \ln\eta^{2}\left(\theta \right)}{\partial \theta^{j}}u^{j}\left(t\right)u^{i}\left(t\right)-u^{i}\left(t\right). \end{array}$$

## 4. Non-Metricity in the α-Geometry

As stated in the Introduction, non-metricity tensor (7) characterizes the deviation from metricity (or metric condition) $\nabla_{i}g_{jk}=0$ in general. Because both curvature and torsion are zero in dually flat spaces in IG, a non-metricity tensor must play a key role. As Hirano and Nagahama [5] pointed out, the non-metricity tensor (12) associated with the α connection $\nabla^{\left(\alpha \right)}$ is proportional to the Amari–Centsov tensor $C_{ijk}\left(\theta \right)$. This can be confirmed by using the definition (13) of the α connection as follows:

$$\begin{array}{cc} \nabla_{i}^{\left(\alpha \right)}g_{jk}\left(\theta \right) & =\partial_{i}g_{jk}\left(\theta \right)-{\Gamma^{(\alpha )\ell }}_{ij}\left(\theta \right)g_{\ell k}\left(\theta \right)-{\Gamma^{(\alpha )\ell }}_{ik}\left(\theta \right)g_{j\ell }\left(\theta \right)\\ \; & =\underset{0}{\underset{︸}{{\overset{\mathrm{LC}}{\nabla }}_{i}g_{jk}\left(\theta \right)}}+\alpha \;C_{ijk}\left(\theta \right)=\alpha \;C_{ijk}\left(\theta \right), \end{array}$$

where metricity ${\overset{\mathrm{LC}}{\nabla }}_{i}g_{jk}\left(\theta \right)=0$ is used.

Generally, an affine connection is characterized by curvature, torsion, and non-metricity, and the coefficients ${\overset{\mathrm{aff}}{\Gamma^{i}}}_{jk}\left(x\right)$ of a general affine connection can be decomposed [24] as

(47) $$\begin{array}{c} {\overset{\mathrm{aff}}{\Gamma^{i}}}_{jk}\left(x\right)={\overset{\mathrm{LC}}{\Gamma^{i}}}_{jk}\left(x\right)+{K^{i}}_{jk}\left(x\right)+{L^{i}}_{jk}\left(x\right), \end{array}$$

where ${\overset{\mathrm{LC}}{\Gamma^{i}}}_{jk}\left(x\right)$ are the coefficients of the Levi–Civita connection, and ${K^{i}}_{jk}\left(x\right)$ denotes a contortion tensor:

(48) $$\begin{array}{c} {K^{i}}_{jk}\left(x\right):=\frac{1}{2}\left\{{T^{i}}_{jk}\left(x\right)-{{T_{j}}^{i}}_{k}\left(x\right)-{{T_{k}}^{i}}_{j}\left(x\right)\right\}, \end{array}$$

with the torsion tensor ${T^{i}}_{jk}\left(x\right):={\overset{\mathrm{aff}}{\Gamma^{i}}}_{jk}\left(x\right)-{\overset{\mathrm{aff}}{\Gamma^{i}}}_{ki}\left(x\right)$, and ${L^{i}}_{jk}\left(x\right)$ denote a *disformation tensor*

(49) $$\begin{array}{c} {L^{i}}_{jk}\left(x\right):=\frac{1}{2}\left\{{Q^{i}}_{jk}\left(x\right)-{{Q_{j}}^{i}}_{k}\left(x\right)-{{Q_{k}}^{i}}_{j}\left(x\right)\right\}. \end{array}$$

The well-known Levi-Civita connection has a non-zero curvature, zero torsion (or torsionless), and zero non-metricity. The Weizenböck connection [25] has a non-zero torsion, zero curvature (or tele-parallel), and zero non-metricity.

We see that non-metricity tensor $Q_{ijk}^{\left(\alpha \right)}\left(\theta \right)$ (12) is totally symmetric when changing indices i,j,k because $C_{ijk}\left(\theta \right):=\partial^{3}\Psi \left(\theta \right)/\partial \theta^{i}\partial \theta^{j}\partial \theta^{k}$. Thus the disformation tensor (49) w.r.t. $Q_{ijk}^{\left(\alpha \right)}\left(\theta \right)$ is

(50) $$\begin{array}{cc} {L^{(\alpha )i}}_{jk}\left(\theta \right) & :=\frac{1}{2}\left\{{{Q^{\left(\alpha \right)}}^{i}}_{jk}\left(\theta \right)-{{{Q^{\left(\alpha \right)}}_{j}}^{i}}_{k}\left(\theta \right)-{{{Q^{\left(\alpha \right)}}_{k}}^{i}}_{j}\left(\theta \right)\right\}\\ \; & =-\frac{1}{2}{{Q^{\left(\alpha \right)}}^{i}}_{jk}\left(\theta \right)=-\frac{\alpha }{2}\;g^{i\ell }\left(\theta \right)C_{\ell jk}\left(\theta \right). \end{array}$$

Consequently, we find that the second term in the (13) coefficients of the α connection expresses the disformation tensor ${L^{(\alpha )i}}_{jk}\left(\theta \right)$. This interpretation gives a physical meaning to the α connection.

### 4.1. Derivation of the Coefficients of the α-Connection

Starting from the non-metricity (12) with the totally symmetric Amari–Centsov tensor (6), we derive the coefficients of the α connection $\nabla^{\left(\alpha \right)}$ in a similar way to obtain the coefficients of the Levi–Civita or Weyl connection.

Since the α connection is torsion-free, as explained in (47), the ${\overset{\left(\alpha \right)}{\Gamma^{i}}}_{jk}\left(\theta \right)$ coefficients are decomposed as

(51) $$\begin{array}{c} {\overset{\left(\alpha \right)}{\Gamma^{i}}}_{jk}\left(\theta \right)={\overset{\mathrm{LC}}{\Gamma^{i}}}_{jk}\left(\theta \right)+{A^{i}}_{jk}\left(\theta \right). \end{array}$$

Here ${A^{i}}_{jk}\left(\theta \right)$ are the components of the unknown tensor we want to obtain and are symmetric when exchanging subscripts, i.e., ${A^{i}}_{jk}\left(\theta \right)={A^{i}}_{jk}\left(\theta \right)$.

Now let us write the non-metricity (12):

(52) $$\begin{array}{cc} \alpha C_{ijk}\left(\theta \right) & =\nabla_{i}^{\left(\alpha \right)}g_{jk}\left(\theta \right)=\partial_{i}g_{jk}\left(\theta \right)-{\Gamma^{(\alpha )\ell }}_{ik}\left(\theta \right)g_{\ell j}\left(\theta \right)-{\Gamma^{(\alpha )\ell }}_{jk}\left(\theta \right)g_{i\ell }\left(\theta \right)\\ \; & =\underset{0}{\underset{︸}{{\overset{\mathrm{LC}}{\nabla }}_{i}g_{jk}\left(\theta \right)}}-{A^{\ell }}_{ik}\left(\theta \right)g_{\ell j}\left(\theta \right)-{A^{\ell }}_{jk}\left(\theta \right), \end{array}$$

where we use the metricity of the Levi–Civita connection $\overset{\mathrm{LC}}{\nabla }$. By permuting indices (i,j,k), we obtain

(53) $$\begin{array}{c} \alpha C_{jki}\left(\theta \right)=-{A^{\ell }}_{ji}\left(\theta \right)g_{\ell k}\left(\theta \right)-{A^{\ell }}_{ki}\left(\theta \right). \end{array}$$

Again permuting the (i,j,k) indices leads to

(54) $$\begin{array}{c} \alpha C_{kij}\left(\theta \right)=-{A^{\ell }}_{kj}\left(\theta \right)g_{\ell i}\left(\theta \right)-{A^{\ell }}_{ij}\left(\theta \right)g_{k\ell }\left(\theta \right). \end{array}$$

By calculating $\alpha (C_{ijk}\left(\theta \right)+C_{jki}\left(\theta \right)-C_{kij}\left(\theta \right))$ by utilizing the torsion-free condition (${A^{\ell }}_{ij}\left(\theta \right)={A^{\ell }}_{ji}\left(\theta \right)$) and from the fact that the Amari-Centsov tensor $C_{kij}\left(\theta \right)$ is totally symmetric, we obtain

(55) $$\begin{array}{c} {A^{i}}_{jk}\left(\theta \right)=-\frac{\alpha }{2}g^{i\ell }\left(\theta \right)C_{\ell jk}\left(\theta \right), \end{array}$$

which is equivalent to the disformation tensor ${L^{(\alpha )i}}_{jk}\left(\theta \right)$ (50). We have derived expression (13) of the α connection from non-metricity (12).

### 4.2. Autoparallel Equations Describing Gradient Flow

Based on non-metricity (12), we can obtain the general autoparallel equations in α geometry

(56) $$\begin{array}{c} u^{k}\left(\lambda \right)\nabla_{k}^{\left(\alpha \right)}u^{i}\left(\lambda \right)=\frac{1}{2u^{2}\left(\lambda \right)}\left(\frac{du^{2}\left(\lambda \right)}{d\lambda }-\alpha C_{jk\ell }\left(\theta \right)u^{j}\left(\lambda \right)u^{k}\left(\lambda \right)u^{\ell }\left(\lambda \right)\right)u^{i}\left(\lambda \right). \end{array}$$

Appendix A provides the derivation of this autoparallel equation. Similarly, we obtain

(57) $$\begin{array}{c} u^{k}\left(\lambda \right)\nabla_{k}^{\left(\alpha \right)}u_{i}\left(\lambda \right)=\frac{1}{2u^{2}\left(\lambda \right)}\left(\frac{du^{2}\left(\lambda \right)}{d\lambda }+\alpha C_{jk\ell }\left(\theta \right)u^{j}\left(\lambda \right)u^{k}\left(\lambda \right)u^{\ell }\left(\lambda \right)\right)u_{i}\left(\lambda \right). \end{array}$$

By using (38) and $u^{2}\left(t\right)=u_{i}\left(t\right)u^{i}\left(t\right)=\eta^{2}\left(\theta \right)$, we have

(58) $$\begin{array}{cc} \frac{du^{2}\left(t\right)}{dt} & =\frac{\partial \eta^{2}\left(\theta \right)}{\partial \theta^{i}}\frac{d\theta^{i}}{dt}=\left(2\underset{u_{i}\left(t\right)}{\underset{︸}{\eta_{i}}}-\underset{C_{ijk}\left(\theta \right)}{\underset{︸}{\frac{\partial g_{jk}\left(\theta \right)}{\partial \theta^{i}}}}\frac{d\theta^{j}}{dt}\frac{d\theta^{k}}{dt}\right)u^{i}\left(t\right)\\ \; & =2u^{2}\left(t\right)-C_{ijk}\left(\theta \right)u^{i}\left(t\right)u^{j}\left(t\right)u^{k}\left(t\right). \end{array}$$

By substituting this relation into the right-hand side of (56) with λ=t and α=−1, it follows that

(59) $$\begin{array}{c} u^{k}\left(t\right)\nabla_{k}^{\left(-1\right)}u^{i}\left(t\right)=u^{i}\left(t\right), \end{array}$$

which is equivalent to the autoparallel Equation (43) describing gradient flow (16). In this way we derived (43) as a special case (α=−1,λ=t) of the general form (56) of autoparallel equations.

In addition, by choosing (α=1,λ=t) and substituting (58) into (57), it follows that

(60) $$\begin{array}{c} u^{k}\left(t\right)\nabla_{k}^{\left(1\right)}u_{i}\left(t\right)=u_{i}\left(t\right), \end{array}$$

which is equivalent to (18) because $u_{i}\left(t\right)=\eta_{i}$ and $u^{k}\left(t\right)\nabla_{k}^{\left(1\right)}u_{i}\left(t\right)=d\eta_{i}/dt$.

Finally, recall that the scalar field $\eta^{2}\left(\theta \right)=u^{2}\left(t\right)$ characterizes the rate of the θ-potential function Ψ(θ), as shown in (23). Differentiating this rate again w.r.t. *t* and using (58), it follows that

(61) $$\begin{array}{c} \frac{d^{2}\Psi \left(\theta \right)}{dt^{2}}=2\eta^{2}\left(\theta \right)-C_{ijk}\left(\theta \right)u^{i}\left(t\right)u^{j}\left(t\right)u^{k}\left(t\right). \end{array}$$

Thus, for the Amari–Centsov tensor $C_{ijk}\left(\theta \right)$, which also characterizes the non-metricity (12) in α geometry, is important to determine the rate of $\eta^{2}\left(\theta \right)=d\Psi \left(\theta \right)/dt$. Note that the relation for $d^{2}\Psi^{⋆}\left(\eta \right)/dt^{2}$ can be obtained in a similar way. Bravetti et al. [26] found, by considering general gradient flow relaxations, that the Amari–Chentsov tensor is crucial for understanding asymmetric speed in relaxations.

## 5. Conclusions and Perspective

We studied some aspects of the fundamental geometrical structures in IG from the perspectives of non-metricity and Weyl’s symmetry. The importance of non-metricity was emphasized. The Amari–Centsov tensor specifies the non-metricty $Q_{ijk}^{\left(\alpha \right)}\left(\theta \right)$ in (12) w.r.t. the α connection $\nabla^{\left(\alpha \right)}$, meanwhile Weyl’s non-metricity in (14) specifies it w.r.t. the Weyl connection $\overset{W}{\nabla }$. The scalar field $\eta^{2}\left(\theta \right)$ in (22) plays a key role hereand characterizes Weyl’s gauge field $\omega^{i}=-\partial_{i}\ln\eta^{2}\left(\theta \right)$, Weyl’s non-metricity $Q_{ijk}^{w}\left(\theta \right)$ in (14), and the rate of the θ-potential function, as shown in (23) during gradient flow (16). Starting from the non-metricity (12) characterized by the Amari–Centsov tensor, we derived the explicit expression of the α connection, for the first time. We showed that, for gradient flow in IG, the variational principle holds based on action $S_{a}$ in (30), which is invariant to local rescaling (31). This enabled us to determine gradient flow in IG as having Hamilton (or Lagrangian) dynamics. In addition, Caticha [27] pointed out that “IG describes the conformal geometry of space”, and it allows an arbitrary choice of the local scale. The arbitrariness of the choice of local scale corresponds to the invariance under the local rescaling (31) in this work. They also pointed out that the distance described in IG, e.g., ds in (28), is *dimensionless*. This means that the parameter space, e.g., θ or η space in IG, is dimensionless, and hence IG has a conformal structure.

Recently, non-metricity formulations of gravity, particularly the Symmetric Teleparallel Equivalent of General Relativity (STEGR) [28] and its extensions of gravity, have attracted attention. As is well-known, general relativity is based on curvature, whereas STEGR is based on non-metricity. We hope our results open the door for the further development of the fundamental structures in IG from the perspective of non-metricity.

## Data Availability

No new data were created or analyzed in this study. Data sharing is not applicable to this article.

## Appendix A. Autoparallel Equations in α Geometry

Here we show the derivation of the autoparallel equations in α geometry based on non-metricity (12) following method similar to that used in [7]. Let us denote velocity $u^{i}\left(\lambda \right):=d\theta^{i}/d\lambda$. We begin with the general form of the autoparallel equations

(A1) $$\begin{array}{c} u^{k}\left(\lambda \right)\nabla_{k}^{\left(\alpha \right)}u^{i}\left(\lambda \right)=f\left(u\right)\;u^{i}\left(\lambda \right), \end{array}$$

where f(u) denotes a certain function of $u^{i}\left(\lambda \right)$ and its derivative $du^{i}\left(\lambda \right)/d\lambda$. We can solve for f(u) as follows: Using non-metricity (12), we have

(A2) $$\begin{array}{cc} u^{i}\left(\lambda \right)\nabla_{k}^{\left(\alpha \right)}\underset{g_{ij}\left(\theta \right)u^{j}\left(\lambda \right)}{\underset{︸}{u_{i}\left(\lambda \right)}} & =u^{i}\left(\lambda \right)\{u^{j}\left(\lambda \right)\underset{\alpha C_{kij}\left(\theta \right)}{\underset{︸}{\nabla_{k}^{\left(\alpha \right)}g_{ij}\left(\theta \right)}}+g_{ij}\left(\theta \right)\nabla_{k}^{\left(\alpha \right)}u^{j}\left(\lambda \right)\}\\ \; & =\alpha C_{kij}\left(\theta \right)u^{i}\left(\lambda \right)u^{j}\left(\lambda \right)+u_{j}\left(\lambda \right)\nabla_{k}^{\left(\alpha \right)}u^{j}\left(\lambda \right). \end{array}$$

Rearranging this relation and contracting with $u^{k}\left(\lambda \right)$ lead to

(A3) $$\begin{array}{c} u^{i}\left(\lambda \right)u^{k}\left(\lambda \right)\nabla_{k}^{\left(\alpha \right)}u_{i}\left(\lambda \right)-u_{j}\left(\lambda \right)u^{k}\left(\lambda \right)\nabla_{k}^{\left(\alpha \right)}u^{j}\left(\lambda \right)=\alpha C_{kij}\left(\theta \right)u^{i}\left(\lambda \right)u^{j}\left(\lambda \right)u^{k}\left(\lambda \right). \end{array}$$

By differentiating $u^{2}\left(\lambda \right)$ w.r.t. λ, we have

(A4) $$\begin{array}{c} \frac{du^{2}}{d\lambda }=u^{k}\left(\lambda \right)\frac{\partial }{\partial \theta^{k}}\left(u^{i}\left(\lambda \right)u_{i}\left(\lambda \right)\right)=u^{i}\left(\lambda \right)u^{k}\left(\lambda \right)\nabla_{k}^{\left(\alpha \right)}u_{i}\left(\lambda \right)+u_{j}\left(\lambda \right)u^{k}\left(\lambda \right)\nabla_{k}^{\left(\alpha \right)}u^{j}\left(\lambda \right). \end{array}$$

where we used

(A5) $$\begin{array}{c} u^{k}\left(\lambda \right)\frac{\partial }{\partial \theta^{k}}=\frac{d\theta^{k}}{d\lambda }\frac{\partial }{\partial \theta^{k}}=\frac{d}{d\lambda }. \end{array}$$

Subtracting (A3) from (A4) and dividing by 2 lead to

(A6) $$\begin{array}{c} u_{i}\left(\lambda \right)u^{k}\left(\lambda \right)\nabla_{k}^{\left(\alpha \right)}u^{i}\left(\lambda \right)=\frac{1}{2}\frac{du^{2}\left(\lambda \right)}{d\lambda }-\frac{1}{2}\alpha C_{kij}\left(\theta \right)u^{i}\left(\lambda \right)u^{j}\left(\lambda \right)u^{k}\left(\lambda \right). \end{array}$$

Contracting (A1) with $u^{k}\left(\lambda \right)$ and comparing it to (A6), we have

(A7) $$\begin{array}{cc} f\left(u\right)\;u^{2}\left(\lambda \right) & =u_{i}\left(\lambda \right)u^{k}\left(\lambda \right)\nabla_{k}^{\left(\alpha \right)}u^{i}\left(\lambda \right)=\frac{1}{2}\left(\frac{du^{2}\left(\lambda \right)}{d\lambda }-\alpha C_{kij}\left(\theta \right)u^{i}\left(\lambda \right)u^{j}\left(\lambda \right)u^{k}\left(\lambda \right)\right). \end{array}$$

Consequently, we obtain

(A8) $$\begin{array}{c} f\left(u\right)=\frac{1}{2u^{2}\left(\lambda \right)}\left(\frac{du^{2}\left(\lambda \right)}{d\lambda }-\alpha C_{kij}\left(\theta \right)u^{i}\left(\lambda \right)u^{j}\left(\lambda \right)u^{k}\left(\lambda \right)\right). \end{array}$$

By substituting this expression into (A1), we obtain (56).
